# Aggressive Versus Moderate Fluid Replacement for Acute Pancreatitis: An Updated Systematic Review and Meta‐Analysis

**DOI:** 10.1002/jgh3.70073

**Published:** 2024-12-13

**Authors:** Muhammad Ehsan, Aamna Badar Ahmad, Haseeba Javed, Afra Zahid, Hafiz Sheheryar Aamir, Huzaifa Ahmad Cheema, Muhammad Ayyan, Biah Mustafa, Abia Shahid, Muhammad Abdullah Ilyas, Kamal Kandel, Rehmat Ullah Awan, Sana Iqbal

**Affiliations:** ^1^ Department of Medicine King Edward Medical University Lahore Pakistan; ^2^ Department of Gastroenterology King Edward Medical University Lahore Pakistan; ^3^ Department of Medicine Kathmandu University Dhulikhel Nepal; ^4^ Department of Internal Medicine Ochsner Health Center New Orleans Louisiana USA; ^5^ Department of Internal Medicine DMC Sinai Grace Hospital Detroit Michigan USA

**Keywords:** acute pancreatitis, fluid replacement, fluid resuscitation, meta‐analysis

## Abstract

**Background:**

A conservative strategy is the primary modality of treatment for acute pancreatitis of which fluid replacement is an important component. Since the results regarding early aggressive versus moderate fluid replenishment for acute pancreatitis are inconsistent, we sought to compare outcomes between the two resuscitation strategies in our meta‐analysis.

**Methods:**

We searched MEDLINE (PubMed), Embase, the Cochrane Library, and ClinicalTrials.gov for all available randomized controlled trials (RCTs) assessing outcomes for patients treated with aggressive fluid replacement compared to moderate fluid replacement. Our primary outcome was all‐cause mortality.

**Results:**

Our meta‐analysis included 6 RCTs involving a total of 632 patients. Our results showed that aggressive fluid resuscitation increased the risk of all‐cause mortality as compared to moderate fluid replacement (RR 2.40, CI: 1.38–4.19). For all of our secondary outcomes which included the development of organ failure, severe pancreatitis, pancreatic necrosis, clinical improvement, development of SIRS, persistent SIRS, and length of hospital stay, the results indicate that there was no significant difference between the two groups.

**Conclusions:**

Aggressive fluid resuscitation is associated with higher mortality as compared to moderate fluid replacement in patients with acute pancreatitis. RCTs with larger sample sizes are needed to provide greater statistical power and establish more definitive conclusions.

## 1 Introduction

Acute pancreatitis can range in severity from a mild, self‐limiting condition to a severe, life‐threatening illness that can cause multiple organ failure. The severity of acute pancreatitis is typically classified using the Atlanta classification system, which divides the condition into mild, moderately severe, and severe categories. The severe forms of acute pancreatitis have a high mortality rate, with estimates ranging from 15% to 40% [1]. Persistent systemic inflammatory response syndrome (SIRS) in response to pancreatic necrosis is responsible for multiorgan dysfunction syndrome (MODS) and death in patients with acute pancreatitis [2].

The management of acute pancreatitis consists of supportive care, including fluid resuscitation, pain management, mobilization, and early enteral nutrition [3]. Treatment with early fluid resuscitation in patients with acute pancreatitis has been found to decrease the occurrence of SIRS and organ failure within 72 h. This is particularly effective in patients with less severe forms of the disease, as opposed to those with severe cases [4]. The current guidelines recommend early aggressive fluid resuscitation [5, 6] but the evidence underlying these recommendations is limited and conflicting [7]. While some observational studies have suggested that hypovolemia is associated with pancreatic necrosis [8, 9], randomized controlled trials (RCTs) investigating early administration of large volumes of fluid have not shown improved outcomes [10, 11].

Previous systematic reviews on this topic have been limited by the small sample sizes and quality of included RCTs, and/or the inclusion of observational studies which confers the risk of confounding bias [12, 13]. Hence, due to availability of new data [14, 15] and inconsistent findings between prior trials, we conducted this meta‐analysis to investigate early aggressive versus moderate fluid resuscitation for the treatment of acute pancreatitis.

## 2 Methods

We conducted this systematic review and meta‐analysis by conforming to the recommendations of the Cochrane Handbook for Systematic Reviews of Interventions [16] and the Preferred Reporting Items for Systematic Reviews and Meta‐Analyses (PRISMA) statement [17]. The protocol was registered with The International Prospective Register of Systematic Reviews (PROSPERO: CRD42023394142).

### 2.1 Eligibility Criteria

**Inclusion Criteria:**

1. Study design: RCTs.
2. Patient population: patients diagnosed with acute pancreatitis.
3. Interventions: early aggressive fluid resuscitation versus moderate fluid resuscitation.

**Exclusion Criteria:**

1. Non‐comparative studies (e.g., case reports, case series) and observational studies.
2. Trials that did not assess any of our pre‐defined outcomes.

### 2.2 Information Sources and Search Strategy

We searched MEDLINE (via PubMed), Embase, the Cochrane Central Register of Controlled Trials (CENTRAL), and ClinicalTrials.gov from inception to July 2023 using a search strategy consisting of keywords and Medical Subject Headings (MeSH) related to “acute pancreatitis,” “fluid resuscitation,” and “fluid therapy.” We did not apply any language, time, or publication restrictions. Other sources included the reference list of relevant articles, which we diligently scrutinized to further retrieve any eligible studies.

### 2.3 Study Selection and Data Extraction

All the literature search results were downloaded to Mendeley Desktop 1.19.8. After the de‐duplication of the articles, two review authors independently performed the screening process based on title and abstracts, followed by the full‐text screening of the articles according to the eligibility criteria. Any disagreement between the two review authors was settled through discussion.

After the process of study selection, the data from included studies were extracted into an Excel spreadsheet. The extracted data included characteristics of included studies (first author, year of publication, and trial design), population (number of patients, gender, and age), intervention and comparators, and the outcome data.

### 2.4 Outcome Measures

Outcomes were compared between the groups undergoing aggressive fluid resuscitation vs. the group which was subjected to moderate fluid resuscitation. Aggressive fluid (Ringer's lactate) resuscitation consisted of a bolus of 20 mL per kilogram of body weight, followed by 3 mL per kilogram per hour. Moderate fluid resuscitation consisted of a bolus of 10 mL per kilogram in patients with hypovolemia or no bolus in patients with normovolemia, followed by 1.5 mL per kilogram per hour [14].

Our primary outcome was all‐cause mortality while our secondary outcomes were the development of organ failure, severe pancreatitis and pancreatic necrosis, clinical improvement, development of SIRS and persistent SIRS, and length of hospital stay.

Severe Acute Pancreatitis (SAP) meant pancreatitis in patients characterized by persistent organ failure (> 48 h) [1]. Pancreatic necrosis was defined as pancreatitis manifesting as necrosis of either the pancreas or the peripancreatic tissue or both [1]. Clinical Improvement within 36 h was defined as the combination of decreased hematocrit, BUN, and creatinine; improved pain; and tolerance of oral diet [18].

### 2.5 Risk of Bias Assessment

The quality assessment of the included studies was carried out by two reviewers independently using the revised Cochrane “Risk of bias” tool for randomized trials (RoB 2.0) [19], which assesses five domains: (1) bias due to the randomization process; (2) bias due to deviations from intended interventions; (3) bias due to missing outcome data; (4) bias in the measurement of the outcome; and (5) bias in the selection of the reported result. Each study was rated as having a “high risk of bias,” “low risk of bias,” or “some concerns.” Any disagreements during the process were resolved through discussion, while a third review author acted as an arbiter when required.

### 2.6 Data Synthesis

We reported dichotomous outcomes using risk ratio (RR) with a confidence interval of 95% and continuous outcomes using mean difference (MD) with a confidence interval of 95%. We performed the meta‐analyses using Review Manager version 5.4 (RevMan). We employed a random effects model to perform meta‐analyses. We assessed the heterogeneity among the studies included in our analysis using the *χ* ^2^ test and the *I* ^2^ statistic. The values of *I* ^2^ statistic were interpreted according to the Cochrane Handbook for Systematic Reviews of Interventions, section 10.10 [16].

To assess the causes of heterogeneity, the subgroup analysis was performed based on age (< 50 years old vs. ≥ 50 years old). A sensitivity analysis was performed by excluding studies with a high risk of bias.

## 3 Results

### 3.1 Study Selection

**FIGURE 1:**
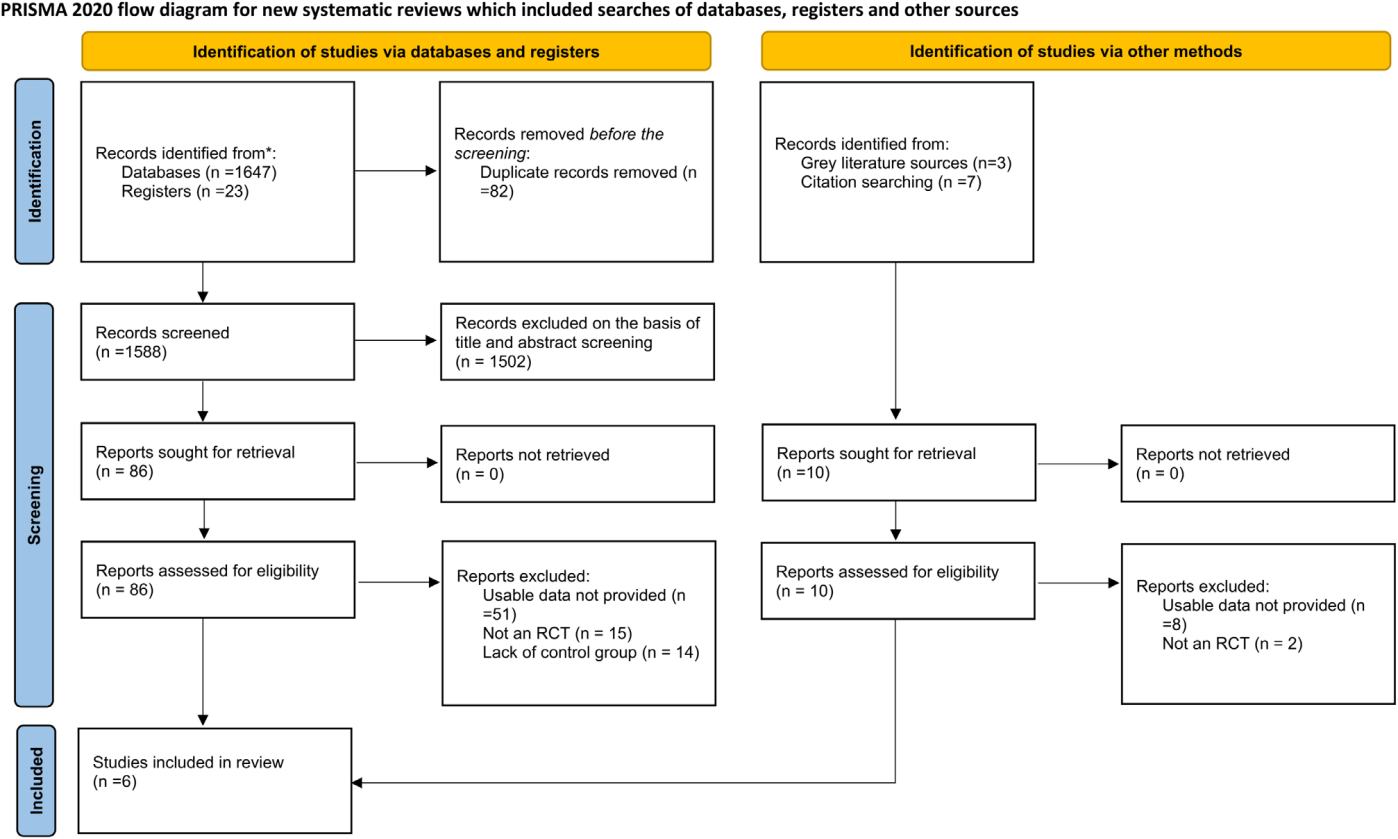
PRISMA 2020 flow chart of included and excluded trials. PRISMA, Preferred Reporting Items for Systematic Reviews and Meta‐Analyses.

A total of 1670 studies were obtained in our preliminary database search. After screening, six RCTs [10, 11, 14, 15, 18, 20] meeting the eligibility criteria were included in our meta‐analysis. Figure 1 shows a PRISMA flowchart outlining the selection procedure.

### 3.2 Characteristics of Included Studies

The included RCTs had a total of 632 patients from seven countries and were published between 2009 and 2022. The replacement fluid used in most studies was Ringer's lactate. Only two trials were conducted blinding of the participants [18, 20]. Aggressive fluid resuscitation was defined as 20 mL/kg bolus followed by 3 mL/kg/h by most studies [14, 15, 18, 20] while moderate fluid resuscitation was defined more variably from 10 mL/kg followed by 1.5 mL/kg/h [14, 18, 20] to 5–10 mL/kg/h [11]. Most of the trials had a follow‐up duration of < 72 h (Table 1) [11, 14, 15, 18, 20].

**TABLE 1:** Study characteristics of individual studies.

Study ID	Country	Year of publication	Age (years)	Female (%)	Trial design	Number of participants	Follow‐up duration	Intervention	Control	Fluid type	Severity of pancreatitis
Buxbaum et al. [18]	USA	2017	44.4 ± 13.7 vs. 45.3 ± 12.3	6 (22) vs. 9 (27)	Single blinded RCT	60 (27 + 33)	36 h	Aggressive (20 mL/kg bolus followed by 3 mL/kg/h)	Standard (10 mL/kg bolus followed by 1.5 mg/kg/h)	Lactated Ringer's solution	Mild acute pancreatitis
Angsubhakorn et al. [20]	Thailand	2021	46.36 ± 14.97 vs. 45.00 ± 15.04	4 (18.2) vs. 6 (27.3)	Single blinded RCT	44 (22 + 22)	36 h	Aggressive = ringers lactate (20 mL/kg bolus followed by 3 mL/kg/h)	Standard lactated ringer sol (10 mL/kg bolus followed by 1.5 mg/kg/h)	Lactated Ringer's solution	Mild acute pancreatitis
Mao En‐qiang et al. [11]	China	2009	51.3 ± 14.3 vs. 50.2 ± 12.0	—	Probably non‐blinded (no info available) RCT	76 (36 + 40)	72 h	Rapid expansion group (fluid rate was 10–15 mL/kg/h)	Controlled expansion group (fluid rate was 5–10 mL/kg/h)	Normal saline and/or ringers lactate as well asplasma, 6% hydroxyethyl starch HES (200 kD/0.5)	Severe acute pancreatitis
Mao En‐qiang et al. [10]	China	2010	49.8 ± 15.4 vs. 48.8 ± 10.1	22 (39.3%) vs. 23 (39%)	Probably non‐blinded (no info available) RCT	115 (56 + 59)	6 months	Rapid hemodilution	Slow hemodilution	Normal saline and/or ringers lactate as well as plasma, 6%hydroxyethyl starch HES (200 kD/0.5)	Severe acute pancreatitis
De‐madaria et al. [14]	India, Italy, Mexico, Spain	2022	56 ± 18 vs. 57 ± 17	68 (55.7%) vs. 59 (46.5%)	Non‐blinded	249 (122 + 127)	72 h	Bolus 20 mL/kg, then infusion 3 mL/kg/h	Infusion 1.5 mL/kg/h, preceded by bolus 10 mL/kg (only if patient has hypovolemia)	Lactated Ringer's solution	Mild acute pancreatitis
Cuéllar‐Monterrubio et al. [15]	Mexico	2020	36.69 ± 15.93 vs. 38.60 ± 15.07	30 (69.7%) vs. 27 (60%)	Non‐blinded	88 (43 + 45)	48 h	Bolus 20 mL/kg, then 3 mL/kg/h for 1st 24 h, then 30 mL/kg/24 h	Infusion 1.5 mL/kg/h for 1st 24 h and subsequently 30 mL/kg/24 h	Hartmann's solution	Mild, moderate and severe AP

### 3.3 Risk of Bias Assessment

Three of the included studies (50%) had an overall low risk of bias. One study (16.67%) had some concerns due to bias arising from the randomization process and bias in the selection of reported results. The remaining two studies (33.33%) were found to be at high risk of bias due to issues in the domain of randomization and selection of reported results (Figure S1) [10, 11].

### 3.4 Effects of Intervention

#### 3.4.1 Primary Outcome

**FIGURE 2:**
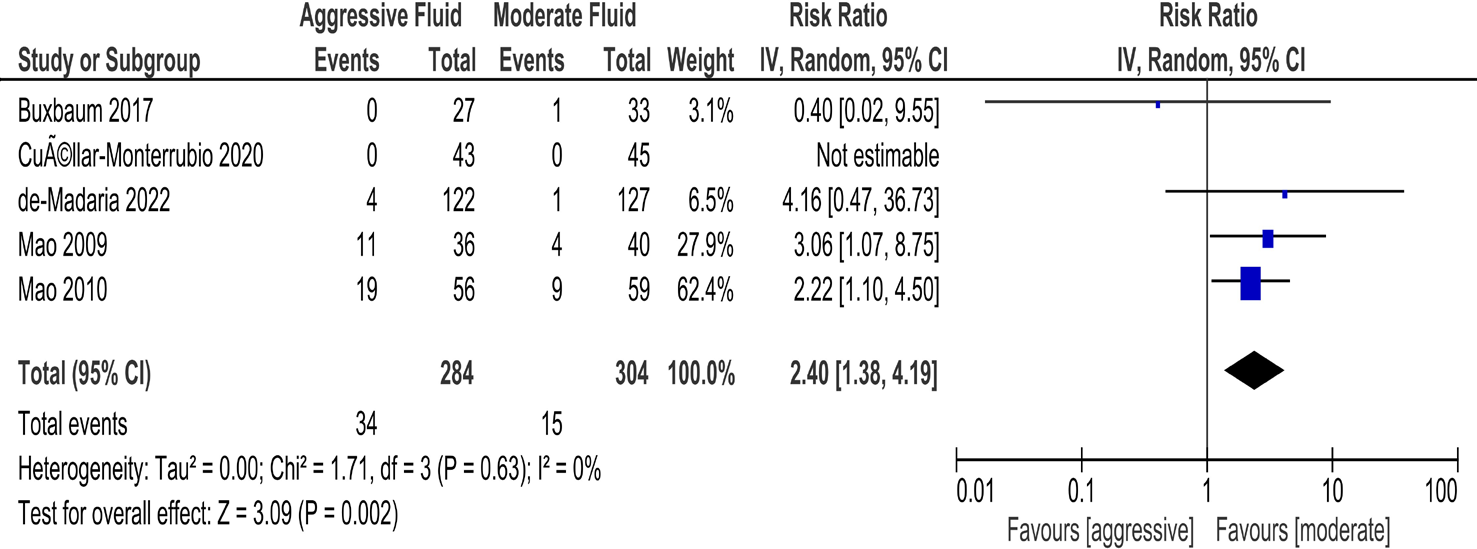
Comparison of mortality between patients receiving aggressive fluid or moderate fluid replacement for acute pancreatitis; IV, inverse variance.

Our meta‐analysis showed that aggressive fluid resuscitation is associated with a higher risk of mortality as compared to moderate fluid replacement (RR 2.40, CI: 1.38–4.19, Figure 2) with minimal statistical heterogeneity (*I* ^2^ = 0%).

On subgroup analysis based on the severity of acute pancreatitis at presentation, there was no significant difference between the subgroups (*p* = 0.77; Figure S2). On subgroup analysis based on age, there was no significant difference between the subgroups (*P*_interaction_ = 0.45; Figure S3).

On excluding two of our studies that were at high risk of bias, our sensitivity analysis yielded no significant difference in mortality between the two groups (RR 1.74, CI: 0.19–15.88, Figure S4).

#### 3.4.2 Secondary Outcomes

##### 3.4.2.1 Development of Organ Failure

Three RCTs reported data on the development of organ failure. Our meta‐analysis concluded that there is no significant difference between the two groups (RR 1.02, CI: 0.53–1.97, Figure 2). Minimal statistical heterogeneity (*I* ^2^ = 4%) was reported.

##### 3.4.2.2 Severe Pancreatitis

Our meta‐analysis found no significant difference between the incidence of severe pancreatitis in patients undergoing aggressive fluid replacement or moderate fluid replacement (RR 1.73, CI: 0.76–3.93, Figure 2). There was minimal statistical heterogeneity (*I* ^2^ = 12%).

##### 3.4.2.3 Pancreatic Necrosis

Meta‐analysis of two studies yielded no significant difference between the two groups (RR 1.82, CI: 0.92–3.59, Figure 2). The estimated heterogeneity was minimal (*I* ^2^ = 0%).

##### 3.4.2.4 Clinical Improvement

There was no significant difference between the two groups in terms of clinical improvement (RR 1.06, CI: 0.73–1.55, Figure 2). Considerable heterogeneity was reported across the studies (*I* ^2^ = 65%).

##### 3.4.2.5 Development of SIRS

There was no significant difference in the development of SIRS between aggressive and moderate fluid replacement strategies (RR 0.67, CI: 0.36–1.24; *I* ^2^ = 2%, Figure 2).

##### 3.4.2.6 Persistent SIRS

We found no significant difference between the two treatment groups in terms of the development of persistent SIRS (RR 1.04, CI: 0.50–2.16; *I* ^2^ = 30%, Figure 2).

##### 3.4.2.7 Length of Hospital Stay

**FIGURE 3:**
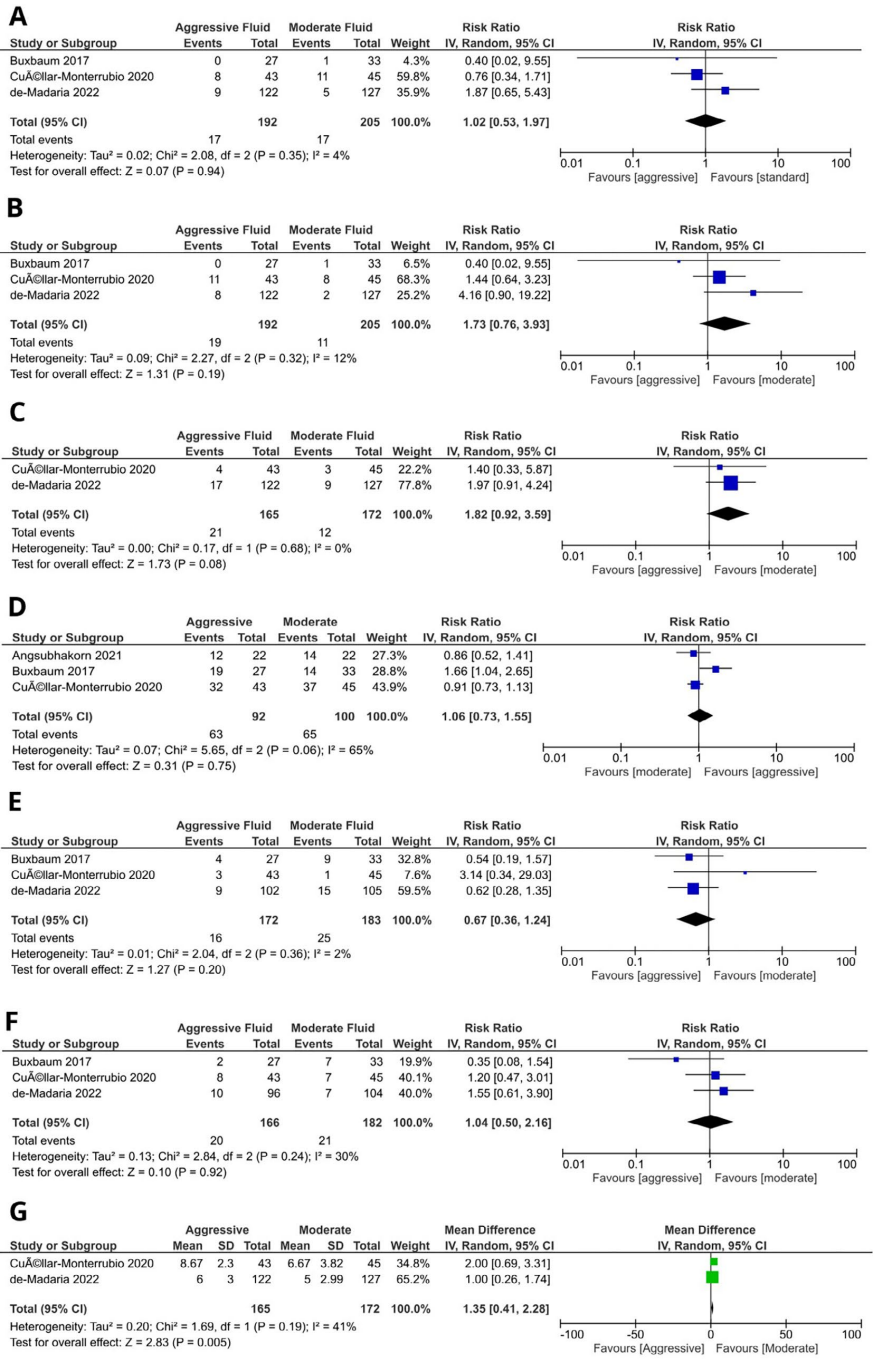
Forest plot for secondary outcomes: (A) Development of organ failure; (B) Incidence of severe pancreatitis; (C) Development of pancreatic necrosis; (D) Clinical improvement; (E) Development of SIRS; (F) Development of persistent SIRS; (G) Length of hospital stay.

Length of hospital stay was reported by two studies. Our meta‐analysis reported no significant difference in the length of hospital stay between the two groups (RR 1.35, CI: 0.4–2.28, Figure 2). Moderate heterogeneity was present between the studies (*I* ^2^ = 41%) (Figure 3).

### 3.5 Discussion

Acute pancreatitis is universally managed initially with fluids, enteral feeding, and continuous monitoring [21]; the most important being fluid therapy. Our meta‐analysis showed a higher risk of mortality in the aggressive fluid replacement group compared to the moderate fluid resuscitation group. There was no significant difference between the two groups with regard to other clinical outcomes. We further stratified our patients based on age since age is an important prognostic factor for acute pancreatitis; however, no significant difference was observed between the two age groups of less than 50 years or older.

Early aggressive fluid therapy has been proposed to improve outcomes as capillary leakage and hypovolemia is a major cause of worse patient outcomes in acute pancreatitis [22]. However, this hypothesis has not been supported by randomized controlled data that has suggested no difference in or even a higher rate of mortality with aggressive fluid replacement, in line with the findings from our meta‐analysis. Although one RCT conducted in mild acute pancreatitis did suggest faster clinical improvement, this was not replicated in our meta‐analysis when pooling results from all trials. The observed lack of benefit from aggressive fluid therapy might be due to the fact that pancreatic necrosis is already present in acute pancreatitis by the time of presentation and diagnosis, and hence, higher fluid volumes cannot prevent necrosis at this stage. In this case, the goal should be to minimize the SIRS response to pancreatic damage and prevent multiorgan damage for which fluid replacement is not the primary treatment modality [23].

Among secondary outcomes including development of organ failure, severe pancreatitis and pancreatic necrosis, clinical improvement, length of hospital stay, development of SIRS, and persistent SIRS, no difference was seen between the aggressive and the moderate treatment groups. Although it is established that aggressive fluid replacement is associated with many adverse effects like acute renal failure and ARDS [12, 24], and it might also be associated with higher CRP levels and hence, worse outcomes [25]. Our results might be explained by the fact that most of these outcomes were only evaluated by a few studies with small sample sizes, and hence lacked statistical power to significant differences. Two trials included patients with severe acute pancreatitis, confounding the effect of therapy and assessment of severe pancreatitis at follow‐up. Further RCTs with larger sample sizes are needed to provide definite conclusions.

Our meta‐analysis compares rates of fluid replacement in patients with acute pancreatitis in a greater cumulative sample size as compared to previous reviews thereby establishing greater confidence in our results [12, 13]. Nevertheless, the pooled number of patients is still low and hence, our findings may suffer from Type II error. Even though most of our included studies were at low risk of bias, they included patients with variable disease severity which may limit the generalizability of our findings.

### 3.6 Conclusions

Our meta‐analysis demonstrated that an early aggressive fluid resuscitation therapy increased the risk of mortality with no difference in other clinical outcomes such as length of hospital stay and development of SIRS, as compared to a strategy of moderate fluid replacement in patients with acute pancreatitis. These results do not support the use of higher volumes of fluid replacement in this patient population; however, further large‐scale RCTs are needed to provide greater statistical power and establish more reliable conclusions.

## Ethics Statement

We confirm that we have read the Journal's position on issues involved in ethical publication and affirm that this report is consistent with those guidelines.

## Conflicts of Interest

The authors declare no conflicts of interest.

## Supporting information


Data S1.

## Data Availability

Data will be provided on reasonable request from the corresponding author.
