# Supplementary material for: Aggressive Versus Moderate Fluid Replacement for Acute Pancreatitis: An Updated Systematic Review and Meta‐Analysis
Source: JGH Open. 2024 Dec 13;8(12):e70073. doi: 10.1002/jgh3.70073 (PMC11645159; doi:10.1002/jgh3.70073)

# Supplementary Material

### Aggressive versus moderate fluid replacement for acute pancreatitis: an updated systematic review and meta-analysis

This supplemental material has been provided by the authors to give readers additional information about their work.

**Supplementary Figure 1.** Quality assessment of randomized controlled trials (RCTs).


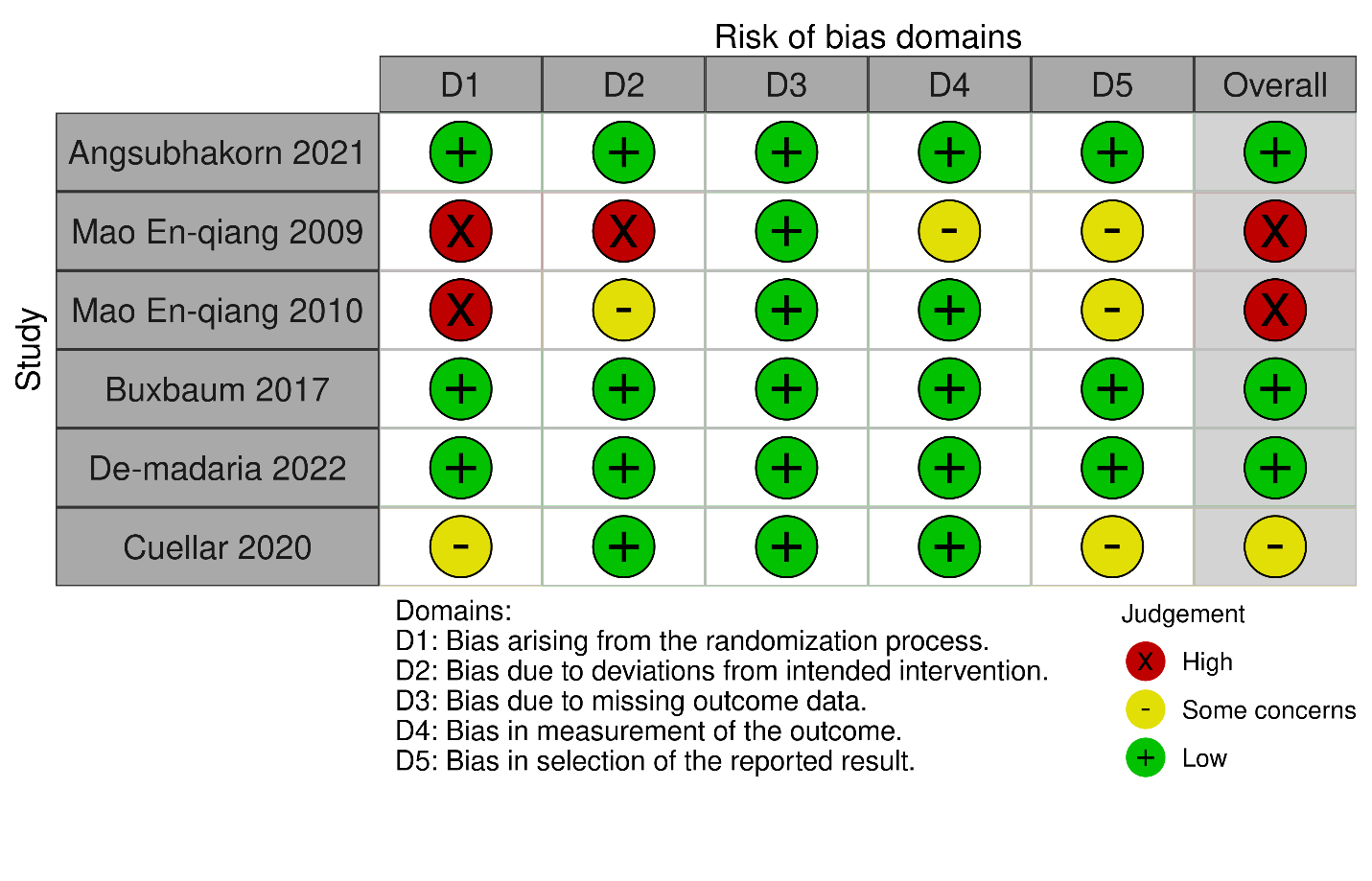


**Supplementary Figure 2. Subgroup analysis of mortality based on the severity of acute pancreatitis at presentation**

**
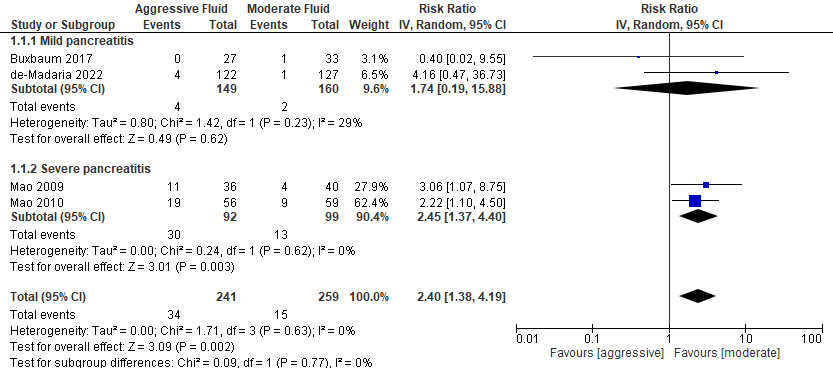
**

**Supplementary Figure 3. Subgroup analysis of mortality between patients receiving aggressive fluid or moderate fluid replacement for acute pancreatitis based on mean age of the patients.**


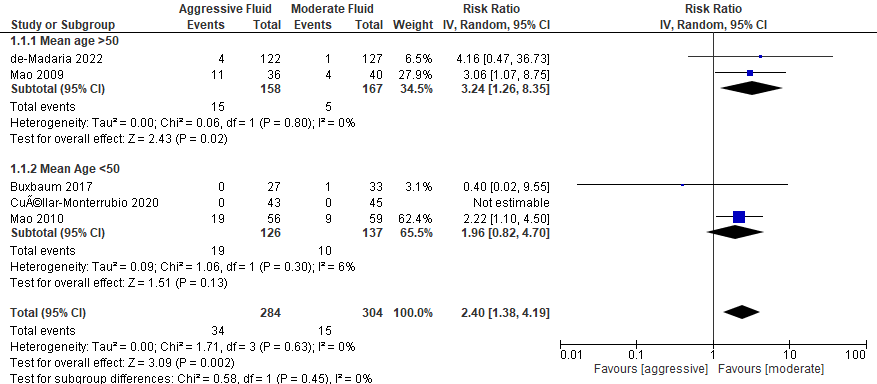


**Supplementary Figure 4. Sensitivity analysis of mortality between patients receiving aggressive fluid or moderate fluid replacement for acute pancreatitis.**


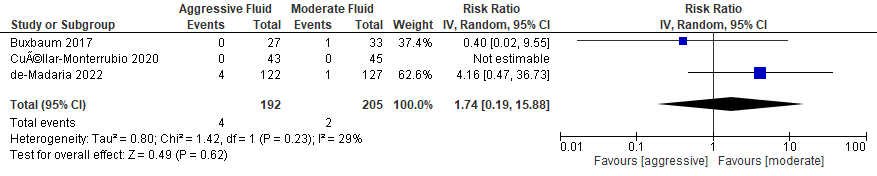

Supplement: Supplementary file 1 — Data S1. [file JGH3-8-e70073-s001.docx]
